# Supplementary material for: Are carbon emissions trading and green financial instruments synergistic? -Comprehensive quantitative research based on content analysis
Source: PLoS One. 2024 Mar 7;19(3):e0298601. doi: 10.1371/journal.pone.0298601 (PMC10919601; doi:10.1371/journal.pone.0298601)
Supplement: S1 Data — (ZIP) [file pone.0298601.s001.zip › Data/地级市Z_F工作报告.zip!/╒π╜¡/╔▄╨╦╩╨/└┤╫╘╣1⁄2╓┌║┼í╛┬φ┐╦╩2╛▌═°í┐_2016.docx]

各位代表：

　　现在，我代表市人民政府，向大会作工作报告，请予审议，并请市政协委员和其他列席人员提出意见。

　　一、“十二五”发展成就和2015年主要工作

　　“十二五”时期是绍兴发展很不平凡的五年。面对错综复杂的宏观环境和艰巨繁重的改革发展稳定任务，市政府全面贯彻国家和省各项方针政策，认真实施省委“八八战略”和市委“重构绍兴产业、重建绍兴水城”战略部署，顽强拼搏、开拓创新、砥砺前行，较好完成“十二五”规划目标任务，在科学发展和现代化绍兴建设道路上迈出了坚实步伐。

　　五年来，我们始终把握发展第一要务，积极应对国际金融危机持续影响，区域综合实力不断提升。预计2015年，全市实现生产总值4470亿元，年均增长8.6%，人均生产总值1.6万美元。一般公共预算收入363亿元，是2010年的1.9倍。完成固定资产投资2580亿元、外贸出口270亿美元、社会消费品零售总额1600亿元，年均分别增长17.7%、5.3%和13.4%。建筑业总产值达到6550亿元，成为全国唯一的建筑产业、住宅产业现代化“双试点”地市。五年间，全市粮食播种面积稳定在218万亩以上、总产量保持在95万吨左右；新增上市企业19家、新三板挂牌企业28家；银行业金融机构由26家增加到42家，存贷款余额分别达到6949亿元和6048亿元，是2010年的1.4倍和1.6倍；滨海新城开发建设全面推进，国家级开发区增加到4家；成功承办第21届中国金鸡百花电影节、第十五届省运会等大型文体活动，获得体育世界冠军3个；高校数量由6所增加到9所、在校生由5.5万人增加到9万人；成功创建全国文明城市，绍兴城市的影响力、美誉度不断提升。

　　五年来，我们始终围绕转变发展方式主线，更加注重质量和效益，经济转型升级持续推进。产业结构不断优化，服务业增加值占生产总值比重年均提高1.3个百分点。“四换三名”、“两化”融合扎实推进，新兴产业投资年均增长26.6%、产值占工业总产值比重由20.4%提高到32.5%。高新技术企业增加到534家，实现高新技术产业产值2350亿元。传统产业改造提升成效明显，累计淘汰4097家企业的落后产能。生态循环农业、观光农业等现代农业蓬勃发展，建成省级现代农业园区98个，粮食生产功能区面积达到78.8万亩。现代金融、现代物流、创意设计等现代服务业日益壮大，“310工程”和“百项百亿”计划顺利实施。实现旅游总收入750亿元，是2010年的2.4倍，兜率天景区、杭州湾海上花田、白塔湖国家湿地公园、绍兴温泉城等项目建成投运。开放型经济发展步伐加快，累计实到外资42亿美元、越商回归投资660亿元，完成境外投资35亿美元。经济交流协作不断深化，援疆、援藏、援青和山海协作任务全面落实。集约发展水平提高，累计盘活存量土地5.3万亩，减少批而未供土地2.3万亩，成为全省唯一的全国国土资源节约集约模范市。节能减排扎实推进，单位生产总值能耗累计下降20%、单位工业增加值用水量下降25%，省下达的化学需氧量、二氧化硫、氨氮和氮氧化物减排目标全面完成。“四化融合、三位一体”安全防控治理体系不断健全，实现安全生产事故起数、死亡人数连年下降。

　　五年来，我们始终坚持统筹区域发展，扎实推进新型城市化，城乡环境面貌逐步改善。城市总体规划获国务院批准，城市发展战略纲要编制完成，城市化率提高到62.5%。部分行政区划调整顺利实施，形成“一市三区”大城市格局，建成高铁绍兴北站、文化中心、科技中心、奥体中心、中国轻纺城体育中心、上虞南北中心大道等一批重大项目。城市管理持续加强，运行机制、考评体系不断完善。县域中心城市加快发展，诸暨城市建成区由47平方公里扩大到55平方公里，嵊新区域协同发展取得积极成效。中小城市分类培育机制不断健全，中心镇权力规制获“中国地方政府创新奖”。美丽乡村“四级联创”成效明显，创建省级美丽乡村先进县5个、美丽乡村示范区20个、美丽农家7万户，农村生活污水治理、生活垃圾集中处理行政村比例分别达到81%和90%。城乡基础设施不断完善，嘉绍大桥、绍诸高速、杭甬客专等建成投用，钦寸水库顺利截流，杭绍台高速控制性节点开工建设，累计新建改造农村公路2375公里，城市日供水能力由80万吨提高到255万吨，城镇污水日处理能力由126万吨提高到193万吨。“811”生态文明建设扎实推进，“五水共治”、雾霾治理、“四边三化”等行动全面开展，IV类以上水质断面比例达到94%，空气质量优良天数占比提高到79.5%，工业污泥、建筑泥浆、工程渣土实现“五统一”管理和市场化运作、资源化利用。累计完成“三改”3433万平方米，拆违3556万平方米。选定香榧为绍兴市树，会稽山古香榧群成功申遗，创建成为国家森林城市。

　　五年来，我们始终突出创新驱动战略，深入推进体制创新、科技创新和社会治理创新，发展动力活力明显增强。开展省级以上改革试点160多项，工业转型升级综合配套改革阶段任务圆满完成，新昌县域科技体制改革、柯桥企业投资项目高效审批、公安车驾管改革在全国全省推广。行政审批制度改革向纵深推进，“四张清单一张网”基本形成，市级部门行政审批事项从843项减少到385项。事业单位分类改革有序开展。经济体制改革不断深化，要素配置市场化改革、企业综合评价分类管理、市场监管体制调整、商事登记制度改革、“营改增”税制改革等顺利实施。国资监管体系不断健全，组建震元健康、文化投资、广电传媒、轨道交通、环境卫生等集团公司。农村综合改革全面推进，农村集体资产股份制改革、农房确权发证基本完成，县级农村产权交易平台实现全覆盖。“八倍增、两提高”科技专项行动扎实推进，发明专利授权量年均增长33%，研究与试验发展经费支出占生产总值比重由1.8%提高到2.3%，连续获评全国科技进步先进市，累计引进人才24.3万人。社会治理不断创新，“枫桥经验”丰富发展，“六五普法”全面完成。　　上接第1版 立体化治安防控体系、公共法律服务体系、大调解工作网络基本建立，流动人口“积分制”管理开始实行，食品药品和农产品质量监管体系不断健全，防灾救灾和应急能力有效增强。社会组织健康有序发展，社会诚信体系逐步完善。

　　五年来，我们始终强化以人为本导向，努力做到民有所呼、我有所应，人民生活更加幸福安康。坚持每年办好一批民生实事，切实解决关系群众利益的热点、难点问题。财政用于民生支出累计达到1187亿元，是“十一五”时期的2.4倍，占同期财政总支出的74%。城镇和农村常住居民人均可支配收入分别达到46836元和25822元，年均分别增长10.3%和11.7%。城镇登记失业率始终控制在3%以内，城乡低保由340元、238元分别提高到623元、544元，最低月工资标准由980元提高到1660元，城乡居民大病保险、残疾人意外伤害保险制度全面实施。养老机构床位增加到3.2万张，是2010年的1.8倍，居家养老服务照料中心实现城市社区全覆盖，成为全国唯一的智慧居家养老服务标准化试点城市，荣获国家“七星级”慈善城市称号。公共文化设施提档升级，文化精品不断涌现。学前教育三年行动计划全面完成，义务教育标准化学校创建率居全省首位，上虞创建成为省教育基本现代化县，镜湖高教园区建成投用。基层卫生和公立医院改革列入国家重点联系市，分级诊疗稳步推行，人感染H7N9禽流感等疫情得到有效防控，人均期望寿命提高到80.4岁。国家卫生城市通过复查。“单独两孩”政策稳步实施。全民健身广泛开展，387所学校体育设施向社会开放。累计新建保障性安居住房10万套，解决城市家庭住房困难7.9万户。智慧城市、光网城市、无线城市建设有序推进，全市城区基本实现光纤到户，人民生活更加便利。

　　五年来，我们始终牢记打铁还需自身硬，着力转变作风、创新治理，服务型政府加快建设。认真贯彻中央“八项规定”精神，深入开展政府系统党的群众路线教育实践活动和“三严三实”专题教育，机关“四风”、“庸懒散奢浮粗”等问题得到有效整改。依法行政理念明显增强，政府法律顾问、决策咨询等制度不断健全，行政问责、审计追责等办法制定实施。人大代表建议、政协提案办理协商更加充分。政府运行机制进一步理顺，全口径财政预算管理全面实施，部门“三公”经费预算基本公开，公车改革有序推进。公务考察、接待、用房和编外人员管理逐步规范，全市“三公”经费支出、因公出国（境）人次比2010年分别下降50.6%和38%。“服务企业、服务基层”工作扎实开展。政府信息公开主动性、广泛性增强，新闻舆论监督不断强化。国防动员、国防教育、民兵、双拥、人民防空、国家安全等工作取得新成绩，军民融合发展成效明显。民族宗教、外事侨务、对台事务、气象、档案、史志、红十字等工作取得新进步，妇女、儿童、老年人保障事业得到新加强。

　　2015年是“十二五”规划最后一年。一年来，全市上下主动适应新常态，积极谋求新发展，预计生产总值同比增长7%，一般公共预算收入增长7.8%，城镇和农村常住居民人均可支配收入分别增长8.5%和9.7%。重点抓了四方面工作。

　　一是标本兼治保稳促调。把稳增长放在突出位置。出台扩大有效投资政策，完善项目推进机制，深入实施“双百双千”工程。创新投融资机制，设立98亿元的产业基金，推行政府和社会资本合作（PPP）模式。调整招商体制，组建内、外资两个招商局，完善招商考核办法。积极应对外贸严峻形势，主动融入“一带一路”，加强政策扶持，落实贸易便利化举措，推进与新加坡等经贸合作。制定拓展消费领域扩大消费需求政策，培育信息、文化、健康等新型消费，积极扶持电商发展，网络零售额增长60%。把促转型作为长期战略。组织实施高端纺织、先进装备制造、绿色化工、金属制造、生命健康、文化旅游、信息经济、现代住建等八大重点产业提升发展工程，建立“八个一”推进机制。积极运用“互联网+”改造提升传统产业，出台时尚产业发展意见，推进印染产业集聚提质，诸暨袜业、新昌轴承列入首批“机器换人”省级试点。加强旅游产业扶持和游线规划，制定促进房地产市场健康发展系列政策，完成服务业重大项目投资265亿元。把帮扶企业作为重要举措。建立实体化帮扶机构，实行企业服务员制度，设立应急转贷基金。组建政府性资产管理公司，多措并举化解银行业不良贷款，多方联手打击逃废债等不法行为。加大支持实体经济力度，地方财政减免税费39.6亿元，争取新增产业项目建设用地指标3550亩，银行贷款投向实体经济比例达到73%。

　　二是做大做强发展平台。把大城市作为承载发展的最大平台，全力推动越城、柯桥、上虞三区融合发展。开展中心城市重大基础设施相连相通三年行动，加快“一环六横八纵”快速路网建设，31省道北延、快速公交五号线等建成通车，孙曹公路袍江和上虞段、104国道柯桥高架等加快建设，城市轨道交通、群贤路东延、绍三线北延等项目前期顺利推进。切实加强三区文明创建、城市管理、社保、教育、卫生等政策措施的统筹衔接。制定嵊新区域协同发展纲要。诸暨城市三环线主线贯通。完善开发区管理体制机制，实行权力下放、充分授权，实施人事和分配制度改革，出台产业集聚、考核激励等系列政策，着力推动开发区提升发展。扎实推进绍兴高新区迪荡湖整治、袍江“两湖”开发、滨海新城中央生态公园建设，优化柯桥、上虞等国家级开发区公建配套设施。加快特色小镇规划建设，黄酒小镇、袜艺小镇列入省级特色小镇首批创建名单，确定绍兴市特色小镇培育对象33个。

　　三是强力治理生态环境。紧紧围绕“誓夺大禹鼎”目标，完善“河长制”责任体系，分阶段开展六大硬仗、十大攻坚、“清三河”达标县创建、夺鼎百日冲刺等专项行动，完成“五水共治”投资189亿元，整治河道305公里、清淤533万立方米，治理垃圾河黑臭河435条、519公里。深入开展排污许可证“一证式”管理、印染废水分质提标集中预处理等全国试点，新增农村生活污水治理受益农户36.8万户。持续推进雾霾治理，加快印染企业“煤改气”步伐，提前实施车用汽油提标升级，推广新能源汽车，淘汰黄标车2.7万辆、燃煤锅炉2885台，PM2.5浓度下降11%。出台“两路两侧”“四边三化”整治方案，完成省定486处广告牌、乱堆乱建等整治任务。开展生活垃圾分类收集试点，启动循环生态产业园建设。实施汤浦水库和曹娥江流域生态补偿。加强环境监管执法，建立环保执法与公检法联动机制，环境违法立案1534起、行政处罚企业1183家（次）。

　　四是认真办好民生实事。市政府承诺的十方面民生实事如期完成。加强创业就业扶持，帮助城镇失业人员再就业4.1万人。启用市民卡，初步实现诊疗、公交、旅游等“一卡通”。启动城镇危旧房改造三年行动，竣工保障性安居住房10765套、分配9975套。优化住宅小区管理，越城区小区移交全面完成。推进公共服务智能化，免费安装智慧居家养老“一键通”8000多户，建成“E邮柜”401个，启用掌上“健康绍兴”医疗服务系统。加强食品安全工作，整治规范小作坊283家、小餐饮5348家，义务教育学校直饮水实现全覆盖。着力缓解城市交通拥堵，深化“四网”建设，创建公交优先示范城市，新增公共自行车5500辆、服务点190个。全面实现绍兴人免费游绍兴。

　　各位代表，过去五年的成绩来之不易。这得益于省委、省政府和市委的正确领导，得益于全市人民的共同努力。在此，我代表市人民政府，向全市人民，向给予政府工作大力支持的人大代表、政协委员、各民主党派、驻绍部队、武警官兵及金融单位，向所有关心、支持和参与绍兴发展的同志们、朋友们，致以崇高的敬意和衷心的感谢！

　　五年来，我们也看到，我市经济社会发展还存在不少问题，政府工作还有很多不足。经济下行压力较大，出口等传统动力弱化，文化旅游等资源优势和消费潜力没有充分释放。经济发展方式仍较粗放，自主创新能力不够强，新兴产业发展不够快，转型升级任重道远。部分企业经营困难，风险隐患仍然存在。大城市竞争力、带动力不够强，三区融合还有很多工作要做。生态环境不容乐观，水、气、土等污染防治还需花更大力气。公共服务均等化水平有待提高，一些山区、老区共享发展还不充分。“四风”问题没有全面根治，有些干部不作为、慢作为，缺乏敢于担当、勇于进取的精神。对这些问题，我们将在今后工作中加倍努力、认真解决。

　　二、“十三五”发展的主要目标任务

　　“十三五”是充满机遇、充满挑战的五年，是绍兴大城市建设的关键期、产业转型升级的攻坚期、高水平全面建成小康社会的决胜期。根据《中共绍兴市委关于制定绍兴市国民经济和社会发展第十三个五年规划的建议》，市政府制定了《绍兴市国民经济和社会发展第十三个五年规划纲要（草案）》，提请大会审议。

　　“十三五”发展的指导思想是：高举中国特色社会主义伟大旗帜，深入贯彻党的十八大、十八届三中、四中、五中全会精神和习近平总书记系列重要讲话精神，认真落实“四个全面”战略布局，深入实施省委“八八战略”和各项决策部署，坚持创新、协调、绿色、开放、共享发展理念，按照“重构绍兴产业、重建绍兴水城”战略部署，以生态文明建设为统领，着眼全市域协同发展，加快发展动力转换，加快产业结构战略性调整，加快中心城市集聚提升和新型城市化步伐，加快社会治理体系和治理能力现代化，不断增强人民群众的获得感、幸福感，高水平全面建成小康社会，为加快建设现代化绍兴奠定坚实基础。

　　“十三五”发展的主要目标是：确保超额完成已经确定的“四翻番”目标，经济年均增长7.5%左右，综合实力全国同类城市排名进一步提升；深入实施“重构绍兴产业、重建绍兴水城”战略部署，建设生态绍兴，共享品质生活，积极构建集约高效生产空间、宜居适度生活空间、山清水秀生态空间，着力提升产业品质、生活品质、环境品质。具体目标是：

　　——产业更具竞争力。产业迈向中高端水平，集群优势更加明显，高端纺织、先进装备制造等更具竞争优势，文化旅游、生命健康等成为支柱产业，新产业、新业态引领作用显著增强，三次产业结构实现由“二三一”向“三二一”转变。

　　——城市更具影响力。中心城市深度融合，诸暨城镇组群集聚辐射能力增强，嵊新城镇组群实现协同发展。城乡发展一体化水平、公共服务均等化水平、区域协调发展水平位居全省全国前列，特色文化、历史文化充分展示。户籍人口城市化率提高5个百分点左右。建成一批宜居宜业宜游的小城市、一批生产生活生态融合发展的特色小镇、一批秀美富裕魅力的美丽乡村。

　　——环境更具吸引力。全面完成节能减排任务，主要污染物排放总量大幅减少，地表水劣V类水质全面消除，全市主要河流、重要水体水质达到或优于Ⅲ类水，PM2.5浓度明显下降，空气质量优良天数占比提高到85%以上，成为国家生态文明建设示范市。

　　——人民更具幸福感。公民文明素质和社会文明程度显著提高。社会保障体系更加健全，教育、文化、卫生等公共服务体系更加完善。城镇和农村常住居民人均可支配收入年均增长8%和8.5%左右，低收入群众收入持续较快增长。社会治理更高效，公民权益和社会公平正义得到切实保障。

　　实现这些目标，必须坚持以生态文明建设为统领，统筹全市域要素资源实现更大发展，厚植特色优势释放发展潜能，强化改革创新增强发展动力，重点做好九方面工作。

　　一是聚焦产业转型升级，重构现代产业新体系。打好转型升级系列组合拳，实施“中国制造2025”绍兴行动计划和“互联网+”行动计划，突出绿色、智能、效益，构建以现代农业为基础、先进制造业和现代服务业为主体、三次产业融合发展的现代产业体系，努力打造转型升级示范区。

　　二是实施创新驱动战略，增强经济发展新动力。积极推进科技创新、开放创新、体制创新、管理创新，实现多轮驱动经济发展。大力培育创新主体，引进创新人才，优化创客服务，全面推动大众创业、万众创新。加快构建以科技城为龙头，科创示范园为主体，科技成果转化站、技术创新点为支撑的科技创新平台体系，加快建成创新型城市。

　　三是提升中心城市能级，构筑协调发展新格局。加快融入“一带一路”、长江经济带，对接沪杭甬都市区，实现配套发展、错位发展和协同发展。推动城乡统筹协调发展，融合大三区、辐射全市域，围绕国家历史文化名城、江南生态宜居水城、长三角区域中心城市定位，努力建设品质之城。

　　四是提高城乡环境质量，建设生态文明新家园。落实最严格的生态保护制度，围绕产业清洁、城市宜居、乡村美丽，深入推进“五水共治”、空气污染治理，积极推行绿色生产生活方式，力争天更蓝、水更清、地更净、山更绿、城更美，全面建设美丽绍兴，成为美丽浙江先行区。

　　五是提升开放发展水平，增创内外开放新优势。坚持内外源并重、内外需共拓、进出口协调、引进来和走出去结合、引资引智并举，全面提高开放水平，以开放带转型、促创新、增活力、提质量。把开发区作为开放主平台，突出主责、创新模式，完善功能定位和产业导向，推动特色化、高端化发展。

　　六是有效增进民生福祉，形成包容共享新局面。坚持以人为本、民生优先，大力发展社会事业，扩大公共服务供给，统筹重大民生政策，健全覆盖城乡的基本公共教育、卫生、文化、养老等均等化服务体系，实现全市省级以上文明城市全覆盖。

　　七是优化完善四大网络，拓展基础设施新空间。加快实施综合交通、能源保障、水利设施、高速信息“四大网络工程”，全力推进杭绍台高速等项目建设，积极做好杭绍台铁路、杭绍甬高速等项目谋划生成，构建形成互联互通、供给高端、系统有效、安全生态的基础设施网络。

　　八是加快文化强市建设，弘扬精神文明新风尚。深入发掘历史文化内涵，着力打响绍兴文化品牌。完善现代公共文化服务体系，提升公益性文化服务水平。努力建成核心价值观深入人心、文化事业繁荣、文化产业发达、文化品牌引领、文化人才辈出、传统文化与现代文明交相辉映的文化强市。

　　九是全面建设法治绍兴，构建社会治理新模式。坚持法治政府、法治社会、法治市场一体化建设，促进治理体系和治理能力现代化。改革完善行政执法体系，加大执纪问责力度，全面提高依法行政能力。创新发展“枫桥经验”，巩固深化“民情通”，牢牢守住稳定、安全底线，切实维护社会公平正义。

　　各位代表，未来五年的新征程已经开启。我们坚信，在中共绍兴市委的正确领导下，经过全市人民的共同努力，“十三五”发展的宏伟蓝图一定能够圆满实现！

　　三、2016年主要工作安排

　　2016年是实施“十三五”规划的开局之年，也是本届政府的收官之年，做好政府工作意义重大。综合分析，建议2016年全市经济社会发展主要预期目标为：生产总值增长7.5%左右；一般公共预算收入增长7%左右；城镇和农村常住居民人均可支配收入分别增长8%和8.5%。重点做好六方面工作。

　　（一）强化创新驱动，加快发展动力转换

　　构建绿色产业体系。深入实施“八大”产业提升发展工程，坚持和完善“八个一”推进机制，努力打造八大千亿级中高端产业集群。以绿色、高端为目标，加快纺织、印染、化工等传统产业改造提升，制订实施“世界领先”的印染产业管控标准，鼓励印染技术创新和品牌创优。做优做强黄酒、丝绸等历史经典产业，支持黄酒产业传承发展和市场拓展。积极培育新兴产业，提高先进装备制造、生命健康等新兴产业比重。大力发展信息经济，推动云计算、大数据、物联网等信息技术创新应用，促进“两化”深度融合。多管齐下整治低小散企业，积极稳妥化解过剩产能，加快淘汰落后产能。创建全国质量强市示范城市，打响“绍兴制造”品牌。推广绿色建筑，新开工建筑产业现代化项目100万平方米。提升发展金融、物流、设计等生产性服务业，支持发展养老、家政、教育等生活性服务业。启动文化旅游业三年推进计划，培育发展动漫影视、创意设计等新兴产业，建设一批文化产业集聚区。深入推广“老绍兴·醉江南”形象品牌，优化绍兴古城、中国兰亭、会稽山、鉴湖·柯岩、曹娥江、大佛寺等旅游功能区布局，整合“时尚金柯桥”“虞山舜水”“西施故里”“唐诗之路”等旅游板块，加快镜湖旅游综合体、金沙东方山水、嵊州飞翼农业休闲小镇等项目建设。积极推动农业现代化，实施农业“两区”绿色发展三年行动计划，开展十大省级生态循环农业示范区创建。培育发展休闲农业、乡村旅游等新兴业态，提升茶叶、香榧、花卉、水果等特色农业效益。强化产业平台支撑，制定实施省级以上功能区提升发展三年行动计划，推动功能区做强特色、差异发展、争先进位。积极创建国家军民融合创新示范区，建设一批军民融合示范企业、产业基地。实行特色小镇三级联创，推进黄酒、袜艺、酷玩、e游、领尚、智造等特色小镇建设。

　　大力推进科技创新。推广新昌县域科技体制改革经验，深入实施科技创新行动计划。深化政产学研合作，促进科技与产业融合。健全多元化科技投入体系，确保研究与试验发展经费支出占生产总值比重达到2.35%。创新高新技术企业培育机制，新增高新技术企业100家、省级科技型企业400家，实现高新技术产业产值2500亿元。完善创新平台体系，规划建设科技城，新建省级以上研发机构40家。积极发展众创、众包、众扶、众筹空间，建设创客示范点20家。

　　深化体制机制改革。加强供给侧结构性改革，着力去产能、去库存、去杠杆、降成本、补短板，增强持续增长动力。建立“四张清单”动态调整机制，提升政务服务网功能，推动政府职能逐步向社会组织转移。加快建立宽进严管、高效便捷的审批体制，建立园区项目集中评价事前审批机制。完善企业综合评价分类管理制度，深化上虞防范企业融资风险体制机制创新。开展诸暨扩大民间投资领域改革试点，推广政府和社会资本合作（PPP）模式。健全国有企业法人治理结构，推进混合所有制改革，加快国有资产证券化步伐。深化上虞上市公司引领产业发展示范区建设试点，鼓励企业上市，力争社会融资总量突破1000亿元，其中直接融资400亿元。

　　释放有效需求潜能。扩大有效投资，启动“十三五”重大项目万亿工程，确保固定资产投资增长10%以上。完善政府投资项目科学决策机制，加强协调保障，提高投资绩效。发挥消费对增长的引领作用，力争社会消费品零售总额增长10%。加快发展服务消费，积极培育信息消费、时尚消费、绿色消费等新增长点，推进线上线下市场互联互通，鼓励商业特色化发展、差异化竞争，提升中国轻纺城、中国领带城等专业市场。大力发展电子商务，推动快递下乡、电商进农村。化解房地产库存，稳定住房消费，保持房地产市场健康发展。

　　切实加强企业服务。完善扶持企业发展的政策体系，打好降低实体经济企业成本“组合拳”。积极引导企业健全现代企业制度，扎实推进“三名”企业培育，推动“个转企、小升规、规改股、股上市”，努力打造一支高素质越商队伍。充分发挥行业协会作用，进一步规范中介服务。健全企业服务员制度，更加真诚、务实、精准帮扶企业。依法推进企业破产重整、兼并重组，健全企业退出机制。支持银行化解不良贷款，打击恶意逃废债和非法集资，确保经济金融安全。

　　（二）强化全市域理念，深入推进城乡统筹

　　优化城乡空间布局。积极推进全市域协同发展，修编城市总体规划，完善市域总体规划、主体功能区规划、土地利用总体规划，逐步实现“多规合一”。加快诸暨城东、城西新城建设。落实嵊新区域协同发展纲要和重大共建项目。实施小城市培育新三年行动计划。完善现代水城规划体系，整治提升“两江十湖”和环城河水环境，构建一批滨水精品游线。加强城乡基础设施建设，做好甬金铁路、杭绍台铁路等项目前期，开工建设杭绍城际铁路等重大工程，加快推进杭绍台高速、800千伏特高压绍兴站等重大项目，力争杭金衢高速改造等工程建成投用。

　　全力推进三区融合。围绕市区同城化、一体化目标，完善促进融合发展的体制机制，统筹制定重要公共政策，统一部署重大公建设施，推进一批交界区域的融合项目建设。修编城市综合交通规划，深入实施重大基础设施相连相通三年行动计划，扎实推进“168”快速路网建设，做好城市轨道交通项目前期工作，规划市区大环线、上虞新港建设。整合三区公交，推进公交一体化。积极推进海绵城市建设，完善城市地下管廊综合规划。加快智慧城市建设，完成智慧健康、智慧交通等18个专项规划，统筹整合视频监控系统等项目建设，提高城市管理科学化、标准化、精细化水平。推动绍兴古城保护发展，进一步理顺体制机制，加强规划管控，加快功能疏解。做好八大历史街区保护和利用，疏浚古城河道，建设慢行系统。积极打造镜湖现代水城示范区，规划建设滨海新加坡小镇，加快袍江“两湖”开发和绍兴高新区迪荡湖建设，促进产城融合。

　　打造美丽乡村升级版。积极推进美丽乡村景观带、美丽乡村公路建设，实施“特色民居”改造工程。扩大农村垃圾减量化、资源化试点范围，确保生活垃圾集中处理行政村比例达到95%。全面完成农村生活污水治理三年行动计划，健全污水处理设施管理机制。稳步推进农村产权制度改革，开展土地承包经营权确权发证，实施嵊州农村承包土地经营权抵押贷款国家级试点，实现三级农村产权交易平台联网运作。规范村级财务管理，力争消除“并村不并账”现象。深化金融支农服务，大力发展民宿经济、家庭农场，多渠道促进农民增收。加大对山区、老区及经济薄弱村、低收入农户的精准扶贫力度。

　　（三）强化绿色发展，建设生态宜居城市

　　健全生态文明制度。完善源头预防、过程控制、损害赔偿、责任追究等生态文明体制机制，严守资源消耗上限、环境质量底线、生态保护红线。健全生态保护补偿机制，深入实施环境损害评估国家级试点。建立排污权交易市场价格形成机制，健全排污权基本账户制度，逐步扩大排污指标市场交易范围。实行最严格的环境监管措施，加密水质、大气、土壤污染防治监测网络，积极开展联合执法、区域执法和交叉执法，以零容忍态度查处环境违法行为。完善反映生态文明建设要求的发展绩效评价体系，构建全方位参与的生态环境社会监督体系。

　　全面开展环境整治。深入推进“五水共治”，坚持和强化“河长制”管理，巩固垃圾河黑臭河整治成果，开展水质竞赛，创建一批生态示范河道，市控以上断面全部消灭劣Ⅴ类水、Ⅲ类以上比例提高10个百分点。大力推进空气污染集中整治，系统治理工业废气、汽车尾气、餐饮油烟、秸秆焚烧和建筑扬尘，加大“煤改气”力度，实施工业锅炉清洁改造，力争空气质量优良天数占比达到81%。加强耕地保护，开展土壤污染治理与修复工作试点，在全市范围内推广工业污泥、建筑泥浆、工程渣土“五统一”管理制度。强势推进“三改一拆”，完成“三改”1000万平方米，拆违500万平方米，完善依法治违长效机制，推动拆后处置项目化，深入开展“无违建县”创建。加强“两路两侧”“四边三化”整治。全面推进平原绿化、林相改造和森林扩面提质，深入实施“八湖”景观工程。

　　加强资源集约高效利用。实施全民节能计划，建立重点企业用能配额核定、增量申购和超额差别收费制度，推广新能源汽车和清洁能源利用，严格控制区域能源消费总量。加强土地利用规划管控和用途管制，深入开展城镇低效用地再开发，实施不动产统一登记发证。加快建设节水型社会。广泛开展节材行动。大力发展生态经济，做好上虞、新昌省级绿色安全制造示范区和试点区工作。扎实推进生活垃圾分类回收和集中处理，加快循环生态产业园建设。

　　（四）强化开放兴市，增强国内国际竞争力

　　统筹发展外贸内贸。加快转变外贸发展方式，努力增加有效出口，实现出口总额增长3%。推进出口企业“双百”培育工程，打造一批外贸综合服务平台，加强重点展会组织引导，巩固扩大欧美日等传统市场，充分拓展东盟、非洲等新兴市场。实施出口品牌战略，培育一批知名品牌和出口精品。探索开展市场采购，推进服务贸易发展基地建设。鼓励企业扩大关键设备、紧缺资源等进口。完善“大通关”机制，申报创建保税物流中心和综合保税区。培育一批内外贸一体化大型企业，支持流通企业与制造企业协同走出去，促进国内外市场互联互通。

　　加大引资引智力度。完善招商引资考核与促进机制，围绕产业强链、补链、建链，紧盯世界500强和央企军企名企，创新以外引外、以民引外、民外合璧办法，力争实到外资9亿美元。实施越商“领头雁”工程，力争越商回归投资250亿元。把利用外资和转型升级相结合，更加注重引进先进装备、技术和管理。深入实施“330海外英才计划”，实行更加开放灵活的人才新政，引进一批创新型领军人才。

　　加强区域合作交流。积极参与义甬舟开放大通道建设，鼓励企业开展跨国并购重组，支持企业到境外承包工程，建设生产基地、营销网络和研发机构。主动参与长三角产业分工，加强与沪杭甬都市区的对接与共享。举办国际友城大会，扩大与新加坡经贸合作，深化与北京西城区战略合作。继续做好山海协作、浙东合作、对口帮扶和援疆、援藏、援青工作。

　　（五）强化为民取向，努力共创美好生活

　　提升社会保障水平。完善就业创业服务体系，统筹做好城镇就业困难人员和农村劳动力转移就业，加快大学生创业园建设。建立全市统一的大病保险制度、市区统一的职工和城乡居民基本医疗保险制度，稳步推进机关事业单位养老保险制度改革，逐步提高最低工资、城乡低保、城乡养老和企业退休人员待遇标准。深化全国智慧居家养老服务标准化试点，逐步把居家养老服务延伸到农村。出台加快残疾人奔小康政策，提高医疗救助、临时救助标准，开展国家基层儿童福利体系建设试点。大力发展红十字、慈善等公益事业。

　　加强社会事业建设。开展“做文明有礼绍兴人”系列活动，巩固深化全国文明城市创建成果。推进现代公共文化服务体系建设，构建图书馆文化馆总分馆机制，深化文化惠民活动。加大地方戏曲扶持力度，实施越剧振兴计划，加强非物质文化遗产保护传承。

　　办好公祭大禹陵、兰亭书法节等重要活动。促进学前教育加快发展、基础教育优质均衡发展，深化新高考配套改革，做好越城、柯桥、诸暨创建省教育基本现代化县工作。加强高校内涵建设，更好发挥高校促进地方发展作用，继续推进绍兴大学创建。加快构建现代职业教育体系，促进产教融合、校企合作。深化基层卫生和公立医院改革，提升发展民办医疗，推进新昌国家卫生县城和嵊州国家卫生城市创建。加强“双下沉、两提升”工作，完善分级诊疗试点。做好人感染H7N9禽流感、登革热等疫病防控。落实“全面两孩”政策。办好国际皮划艇马拉松等赛事，开放利用公共体育场地设施，推动全民健身。

　　创新社会治理体系。坚持和发展“枫桥经验”，完善立体化治安防控体系，深化信息化实战警务，全力做好G20峰会相关工作。加强乡村治理，深化“枫桥式”镇街、民主法治村社创建。推进“七五”普法。加快社会信用体系建设，搭建公共信用信息平台。稳步实施户籍制度改革，深化流动人口“积分制”管理，逐步建立以居住证制度为基础的服务保障体系。整合优化各类载体，建设统一政务咨询投诉举报平台。开展企业与职工“双爱”活动，构建和谐劳动关系。强化食品药品安全监管，加强公共应急和气象等防灾减灾体系建设。全面开展安全生产“企业主体责任落实年”和“行政执法规范建设年”活动，确保安全生产事故起数、死亡人数继续下降。支持军队和武警部队建设与改革，继续做好国防动员、国防教育、人民防空和双拥优抚、国家安全等工作。发展民族宗教、外事侨务、对台事务等事业，支持工会、共青团、妇联、科协等开展工作。

　　办好十方面民生实事。（1）全力治污水，完成河道整治180公里、清淤600万立方米。加强农村饮水安全保障，改善3万农村人口饮水条件。（2）深入治雾霾，启动热电厂超低排放改造，淘汰燃煤小锅炉500台，全面淘汰黄标车。（3）加强养老服务体系建设，新增居家养老服务照料中心500家，实现城乡社区全覆盖。新增机构养老床位2300张。（4）加大城市治堵力度，新增公交车100辆、新（改）建公交站点100个，新增公交运营里程100公里。（5）实施农村惠民交通工程，提升改造农村公路200公里，完成路面大中修280公里，实施安保工程100公里，新（改）建港湾式停靠站300个。（6）完善公共服务体系，新建（扩建）幼儿园50所，全科医生规范签约服务100万人，新增农村文化礼堂100个。（7）加大就业扶持力度，新增城镇就业10万人，帮助城镇失业人员再就业3.5万人，培养高技能人才2万人。（8）加强“菜篮子”工程建设，新建“菜篮子”基地1000亩、改造提升1500亩。加强农产品质量安全保障，“双低”农药应用实现全覆盖，农产品合格率达到98%。（9）拓展市民卡在教育管理、图书借阅等方面的应用，新增服务网点150个，发卡超过150万张。（10）持续推进三区交通融合，建成二环北路拓宽、孙曹公路袍江和上虞段等工程，加快群贤路东延、绍三线北延、104国道柯桥高架等重大项目建设。

　　（六）强化自身建设，提升政府治理水平

　　加快建设法治政府。深入推进依法行政，全面落实法治政府建设实施纲要。自觉接受人大依法监督、政协民主监督，支持司法机关依法开展工作。加快推进地方立法，健全与市人大的立法衔接机制。加强法律顾问队伍建设，坚持政府集体学法制度。规范重大行政决策程序，健全公众参与、风险评估、合法性审查等制度，提高科学决策水平。严格行政规范性文件制定程序，加强备案审查。深化简政放权、放管结合、优化服务等政府自身改革，实现乡镇（街道）权力清单和责任清单全覆盖。实施综合执法体制改革，推动执法重心下移。

　　着力优化机关作风。巩固党的群众路线教育实践活动和“三严三实”专题教育成果，完善领导干部社会评价制度，推动改进作风常态化、长效化。牢固树立创先争优理念，积极搭建比学赶超平台。大力弘扬敢于担当精神，探索完善鼓励创新、宽容失败的体制机制，为敢于担当的人担当，努力营造干事创业的良好氛围。推行重点工作清单化管理，着力强化抓落实导向。严格公务员考核，加大行政问责力度。

　　健全权力监督体系。加强行政监察、审计监督和专项巡查，规范重点工程、重大项目和公共资源交易统一平台建设。深入推进政务公开，实现财政预决算向社会全公开。加快建立现代财政制度，开展财政专项资金第三方评价，强化财政资金绩效管理。深化国库集中支付和公务卡改革，探索将政府性投资项目纳入国库集中支付范围。完善督查机制，实行进度通报、信息反馈、对账销号和舆论推动等制度，确保各项任务落地生根。认真贯彻廉洁从政各项规定，健全廉政风险防控机制，全面整治庸政懒政怠政，着力打造一支忠诚干净担当的干部队伍。

　　各位代表，干在实处永无止境、走在前列要谋新篇。让我们更加紧密地团结在以习近平同志为核心的党中央周围，在中共绍兴市委的坚强领导下，坚定信心、苦干实干，合力谱写新篇章，接力创造新辉煌，为高水平全面建成小康社会、加快建设现代化绍兴而努力奋斗！
